# Competition, seed dispersal and hunting: what drives germination and seedling survival in an Afrotropical forest?

**DOI:** 10.1093/aobpla/plz018

**Published:** 2019-03-28

**Authors:** Ola Olsson, Gabriela Nuñez-Iturri, Henrik G Smith, Ulf Ottosson, Edu O Effiom

**Affiliations:** 1 Department of Biology, Biodiversity Unit, Lund University, Ecology Building, Lund, Sweden; 2 Herbario MOL de la Facultad de Ciencias Forestales, Universidad Nacional Agraria La Molina, Lima, Peru; 3 A.P. Leventis Ornithological Research Institution, University of Jos, Jos, Nigeria; 4 Cross River State Forestry Commission, Calabar, Nigeria

**Keywords:** Competition, plant-animal interactions, seed-dispersal, seed-germination, seed-predation

## Abstract

Disentangling the contributions of different processes that influence plant recruitment, such as competition and seed dispersal, is important given the increased human-mediated changes in tropical forest ecosystems. Previous studies have shown that seedling communities in an Afrotropical rainforest in southeastern Nigeria are strongly affected by the loss of important seed-dispersing primates, including Cross River gorillas (*Gorilla gorilla diehli*), chimpanzee (*Pan troglodytes elioti*) and drill (*Mandrillus leucophaeus*). Here we study how germination and survival of tree seedlings are affected by competition and reduced seed dispersal in three contiguous forest reserves, in southeastern Nigeria, with similar mature tree species composition and structure. We use an experimental design aimed at manipulating the effect of competition among seedlings in three protected and three hunted sites within the reserves. We use a total of sixty 5 × 5 m plots of three types: plots cleared of all seedlings, plots selectively cleared of all primate-dispersed seedlings and control plots. All seedlings were identified, measured, assigned to dispersal mode and tagged, and after 1 year we evaluated survival, mortality and new recruits. We found that in hunted sites germination of abiotically dispersed species was over four times higher in cleared plots compared to control plots, whereas germination of primate-dispersed species was the same, which indicated that dispersal limitation was the dominant force in seedling recruitment in hunted sites. This was supported by the fact that the germination of all dispersal modes in the selectively cleared plots in protected sites was similar to the control plots in the same sites, but germination of abiotically dispersed species was significantly lower than in cleared plots in hunted sites. Competition among seedlings was mostly evident from the fact that 75 % more seedlings of primate-dispersed species germinated in cleared compared to control plots in protected sites. We conclude that inter-seedling competition may be irrelevant to seedling recruitment in hunted sites, where dispersal limitation appears to be a much stronger force shaping the seedling plant community, and thus hunting indirectly reverses the importance of competition and dispersal limitation in structuring seedling communities.

## Teaser

The dynamics in the seedling community in the forest understory play a central role in directing plant recruitment and the future composition and structure of the forest. In tropical forests large mammals have an important role as seed dispersers, but many of these mammals are threatened by human activities, including hunting. This experimental study in Nigerian rainforests reveals that hunting indirectly reverses the importance of inter-seedling competition and dispersal limitation. Thus, the loss of large mammals, such as apes and monkeys, is likely to change the future of the forests.

## Introduction

Hunting in otherwise intact tropical forests removes organisms from some trophic levels, which can change population and community dynamics at other levels (Dirzo and Miranda 1991; Terborgh *et al.* 2001; Fa and Brown 2009; Lambert 2011; Effiom *et al.* 2013, 2014). The preferred game species can be dispersers of seeds (e.g. primates, large birds), predators of seeds (e.g. rodents), browsers of juvenile plants (e.g. ungulates) or disturbers of the forest floor (e.g. pigs or peccaries) (Redford 1992; Chapman and Onderdonk 1998; Andresson Djurfeldt *et al.* 2017; Nunez-Iturri *et al.* 2008). Hunting one or more of these groups will change animal abundances through direct and indirect effects (Wright 2003) and this may change seed dispersal patterns and seed or seedling survival in the forest (Effiom *et al.* 2014; Beaune 2015; Culot *et al.* 2017). It has previously been shown that this has effects on the densities and richness of the seedlings on the forest floor (Wright 2003; Nuñez-Iturri and Howe 2007; Terborgh *et al.* 2008; Culot *et al.* 2017), which eventually could lead to changes in the mature forest tree composition and structure.

In a previous study in African rainforests (Effiom *et al.* 2013), we found that the regenerating cohorts of plants differed strikingly between forests protected from hunting and forests with hunting. In the protected forests seedlings of primate-dispersed species were more common, just as with the mature trees. Least common were abiotically dispersed species, and species dispersed by other animals were intermediate. In the hunted forests these patterns were reversed. The changes of the regenerating plant community were correlated with the distortion of the mammal community caused by hunting: seed dispersers were less common and seed predators more common in hunted forests (Effiom *et al.* 2013, 2014). That is, the protected sites have a more or less intact seed disperser community, with primates including the Cross River gorilla (*Gorilla gorilla diehli*), chimpanzee (*Pan troglodytes elioti*), drill (*Mandrillus leucophaeus*) and several species of smaller monkeys including putty nosed monkey (*Cercopithecus nictitans*) and Mona monkey (*C. mona*), whereas the hunted sites are practically empty of these animals (Effiom *et al.* 2013, 2014). The seed predators, such as large rodents, are more common in hunted sites (Effiom *et al.* 2013, 2014). However, while the patterns are clear, the underlying ecological processes are less well known (Stoner *et al.* 2007; Vanthomme *et al.* 2010; Effiom *et al.* 2014; Rosin and Poulsen 2016).

Seedling establishment depends on seed survival and germination, both of which are influenced by dispersal (Moles and Westoby 2004; Terborgh *et al.* 2011), and undispersed seeds may have lower establishment success due to density-dependent seed mortality, and increased competition (Janzen 1970; Connell 1971; Terborgh 2013). Certain functional traits, including seed size, are thought to affect seedling germination, densities and survival in the shaded forest understory (Paz and Martínez-Ramos 2003; Moles and Westoby 2004). However, species with large seeds are likely to suffer high seed mortality in areas with insufficient seed dispersal as aggregated undispersed seeds attract vertebrate and invertebrate seed predators as well as pathogens (Augspurger 1984; Chapman and Chapman 1995; Terborgh *et al.* 2011). Seed size also has profound effects on fecundity, successional dynamics, persistence in the seed bank, establishment success, seedling survival, seedling growth rate and competitive ability among species (Connell and Slatyer 1977; Rees *et al.* 2001). For example, plant species with smaller seed size and higher fecundity are better dispersers and colonizers and may be favoured in defaunated forests where large-seeded mammal-dispersed species are disadvantaged in the absence of their disperser. On the other hand, large-seeded plants are often fecundity limited but may be stronger competitors which enhances establishment success (Crawley 1990; Tilman 1994).

Competition is widely believed to play a crucial role in structuring plant communities (Goldberg and Barton 1992). However, in the last decades it has been argued that competition may be irrelevant in controlling seedling regeneration in stressed hyper-diverse tropical forests where resource limitation and/or natural enemies may be more important for influencing seedling germination and survival (Paine *et al.* 2008; Svenning *et al.* 2008; Terborgh *et al.* 2011).

In this paper, we aim to improve our understanding of the ecological processes that lead to changes in seedling communities following the decimation of seed-dispersing mammals in hunted but otherwise intact forests (Effiom *et al.* 2013). We therefore assessed germination and survival of seedlings in hunted and protected tropical forest sites in southeastern Nigeria, in an experimental design to create different levels of competition. It is important to bear in mind that what we term germination is in fact the product of at least four processes: seed rain, seed predation, germination and survival during the study year. The idea of the experimental treatment is to reduce competition for space and resources between seedlings to a minimum, but will not allow us to separate between competition for space or resources. We hypothesize that germination of primate-dispersed species should be related to the density of seed dispersers in the forest (i.e. hunting), and that primate-dispersed seedlings secondarily affect the regeneration of other seedlings through competition.

## Methods

The study was performed in three contiguous forest reserves in Cross River state, Nigeria (6°10′N, 9°0′E). The reserves are Cross River National Park (CRNP), Mbe Mountain Community Wildlife Sanctuary (MMWS) and Afi Mountain Wildlife Sanctuary (AMWS). These reserves have areas where hunting pressure from adjacent villages is low and areas where protection from hunting is insufficient, resulting in significantly lower densities of primates and higher densities of seed predators in the hunted parts (Effiom *et al.* 2013, 2014; Andresson Djurfeldt *et al.* 2017). We set up our study so that in each of the three reserves we had one site in a protected area and one site in an area affected by hunting, totalling three protected sites and three hunted sites. The forests in these reserves are tropical, evergreen lowland rainforest, at altitudes ranging from 150 to 800 m above sea level. Forest structure and tree species composition were similar between hunted and protected sites, and the study sites were not affected by any kind of logging (Effiom *et al.* 2013). As mentioned above, protected areas have practically intact mammal communities, whereas hunted sites have significantly lower densities of primates and higher densities of large rodent seed predators (Effiom *et al.* 2013, 2014).

We studied germination and survival in a total of sixty 5 × 5 m plots randomly placed along four 1-km transects in each site. The plots were of three kinds: at each transect we had one control plot (4 per site, 24 in total) and one which was cleared of all seedlings (≤1 m tall; 4 per site, 24 in total), in order to study germination in the absence of competition from other seedlings. These plots were established in the wet season in 2009 in CRNP and AMWS, and in the wet season 2010 in MMWS. After 1 year we identified, counted and tagged all individuals that had germinated, and at the same time we established control plots, in which we individually identified and tagged all standing seedlings in the wet season, to be able to study both germination and mortality until the following year, under normal competition. Finally, we established another set of experimental plots called the selectively cleared plots (4 per protected site, 12 in total), in which we removed all primate-dispersed seedlings, in order to study germination and mortality in the absence of competition from primate-dispersed species. This was done to mimic the competitive regime found in hunted sites. All plots were placed randomly along the transects, with the restriction that all were placed under closed canopy, avoiding gaps and thus making sure light conditions were similar (Effiom *et al.* 2013).

Seedlings were identified to species, and the species were classified by dispersal mode, as described in Effiom *et al.* (2013). The dispersal modes used are primate dispersed, dispersed by other animals, and abiotically or vegetatively dispersed.

### Statistical analyses

To analyse germination we used the numbers of new plants appearing in the plots 1 year after the first visit. Residuals were overdispersed when fitting Poisson models, but not when fitting negative binomial models, and therefore we used the latter kind with a log link function. In all cases model fit was much better for the negative binomial model (10 AIC, Akaike Information Criterion, units or higher improvement). The response variable was the pooled number of seedlings in each dispersal mode (abiotically dispersed, primate dispersed and dispersed by other animals) within each plot, and we used a nested random structure to represent the experimental design with dispersal modes within plots, within transects, within sites, and as fixed factors we used plot treatment, dispersal mode and hunting level. Statistical analyses were performed using R 3.5.1 (R-Core-Team 2018), with the package *lme4* (Bates *et al.* 2015), function glmer.nb.

To analyse survival we noted which tagged standing seedling individuals remained alive or had died in the plots 1 year after the first visit. Here we assumed binomial error distribution and used a logit link to analyse the effect of treatment. We used the same nested random structure as above, but additionally added a random intercept term for species, and used plot treatment, dispersal mode and hunting level as fixed factors.

For all models we used likelihood ratio tests to evaluate the effects of fixed factors, and present likelihood ratio χ^2^-values and associated *P*-values. We only present these values for the highest order significant terms, i.e. not for main effects in a model with significant interaction terms. In the analysis of the complete clearing treatment the three-way interaction between hunting, plot treatment and dispersal mode was significant, and therefore we sliced the data set by plot treatment and by hunting level to allow inferences on more interpretable two-way interactions. Inferences are based on the above-mentioned significance tests, and on plots of estimated marginal means and associated 95 % confidence intervals, which were calculated using the *effects* package in R (Fox 2003). Residuals were inspected graphically, and found to conform well to model assumptions.

Data are available at datadryad.org with a DOI: 10.5061/dryad.48b2dd0.

## Results

### Germination

**Figure 1.**
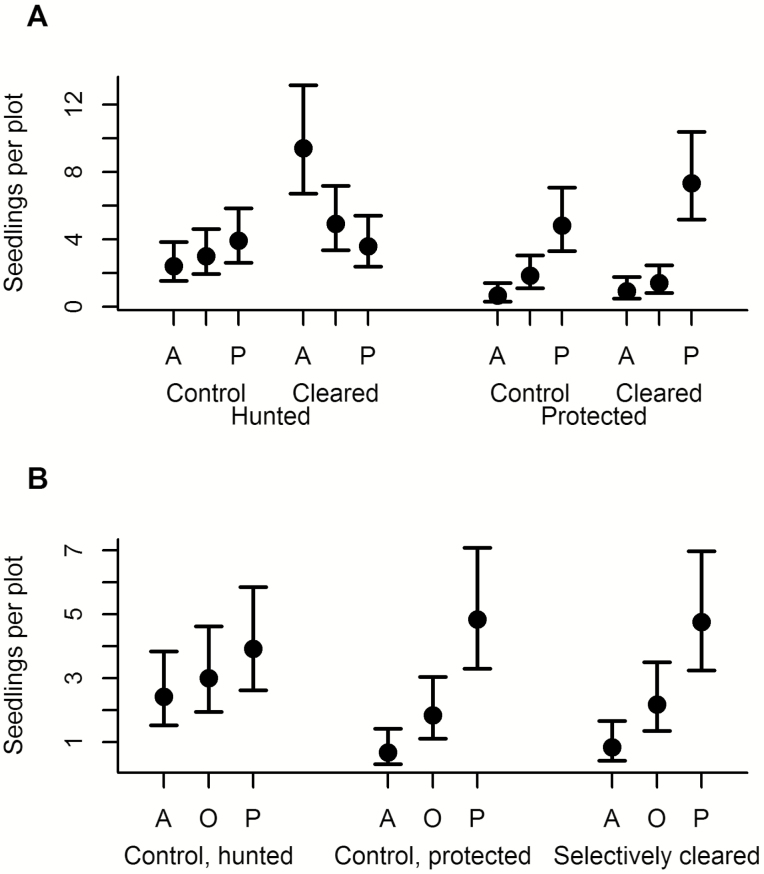
(A) Number of germinating seedlings in hunted and protected sites and in control and completely cleared plots. A represents abiotically dispersed seedlings, P represents primate-dispersed seedlings, while O are seedlings dispersed by other animals rather than primates. The dots are the estimated marginal means from analyses, and bars represent the 95 % confidence intervals of these means. That is, two groups are significantly different if the mean of one group falls outside of the interval of another group. (B) Number of germinating seedlings in selectively cleared plots located in protected sites, and in the control plots in hunted and protected sites (same as in Fig. 1A).

The complete clearing treatment had different effects on germination on the three dispersal modes used, depending on hunting. That is, in the analysis of germination the three-way interaction between hunting, plot treatment and dispersal mode was significant (χ^2^ = 6.49, df = 2, *P* = 0.039; Fig. 1A). To tease this interaction apart, we sliced the data set both by plot treatment and by hunting level to test specific hypotheses.

In cleared plots in hunted sites abiotically dispersed seedlings were most common, and primate-dispersed seedlings least common. This pattern was opposite to, and deviated significantly from, both the control plots in the same sites (interaction between dispersal mode and plot treatment in hunted sites: χ^2^ = 9.69, df = 2, *P* = 0.008; Fig. 1A), and the cleared plots in protected sites (interaction between dispersal mode and hunting level in cleared plots: χ^2^ = 16.94, df = 2, *P* = 0.0002; Fig. 1A).

Furthermore, in control plots, abiotically dispersed species were over three times as common in hunted sites compared to protected sites (interaction between dispersal mode and hunting level in control plots: χ^2^ = 8.75, df = 2, *P* = 0.013; Fig. 1A).

However, in the protected sites there was no difference in germination between the dispersal modes between cleared and control plots (interaction between dispersal mode and plot treatment: χ^2^ = 2.83, df = 2, *P* = 0.2). Also, the overall numbers of seedlings germinating did not differ between cleared and control plots (main effect of plot treatment: χ^2^ = 1.94, df = 1, *P* = 0.16), but the number of germinating primate-dispersed seedlings was significantly higher than the other dispersal modes (main effect of plot treatment: χ^2^ = 52.7, df = 2, *P* < 0.0005; Fig. 1A).

The selectively cleared plots in protected sites were similar to the control plots in the same sites, whereas they differed from control plots in hunted sites, where in particular germination of abiotically dispersed seedlings was higher (treatment by dispersal mode interaction: χ^2^ = 10.6, df = 4, *P* = 0.031; Fig. 1B).

### Survival

**Figure 2.**
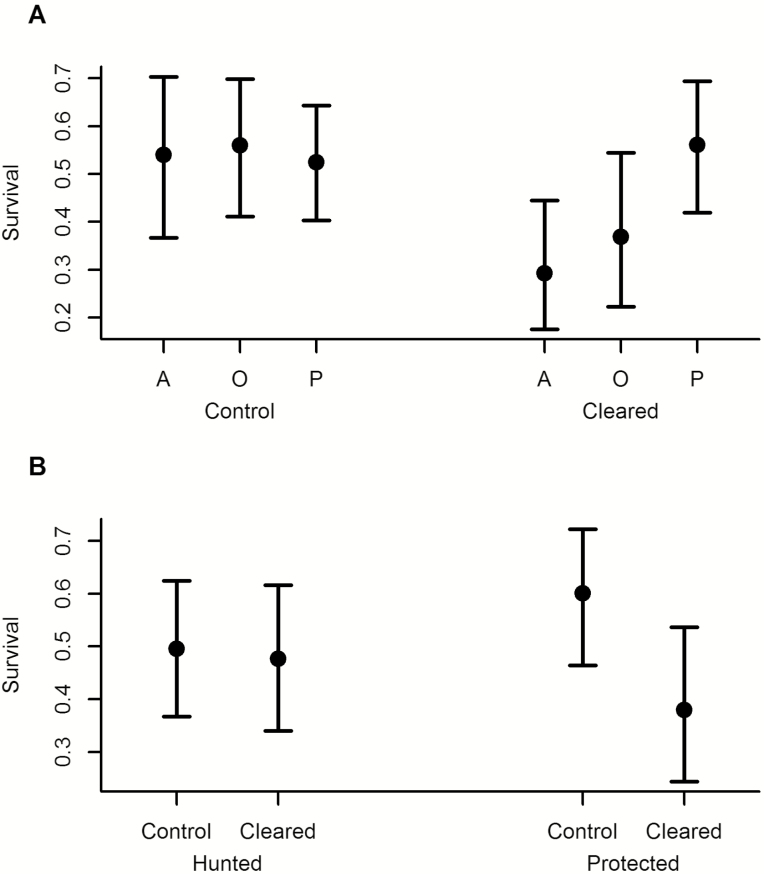
(A) Survival rates of seedlings of the three dispersal modes in control and cleared plots. The dots are the estimated marginal means from analyses, and bars represent the 95 % confidence intervals of these means. (B) Survival of seedlings in control and cleared plots, in hunted and protected sites.

In completely cleared plots there was higher survival among primate-dispersed seedlings than among the other two dispersal modes, but in control plots there was no difference among the dispersal modes (treatment by dispersal mode interaction: χ^2^ = 8.03, df = 2, *P* = 0.018; Fig. 2A). In protected sites, seedlings had higher survival in control plots than in cleared plots, but in hunted sites there was no difference (treatment by hunting interaction: χ^2^ = 4.77, df = 1, *P* = 0.029; Fig. 2B). Neither the interaction between dispersal mode and hunting (χ^2^ = 2.38, df = 2, *P* = 0.3), nor the three-way interaction (dispersal mode by hunting by treatment: χ^2^ = 1.05, df = 2, *P* = 0.6) were significant.

The selectively clearing experiment had no effect on survival (treatment by dispersal mode interaction: χ^2^ = 3.72, df = 4, *P* = 0.4; main effect of dispersal mode: χ^2^ = 0.37, df = 2, *P* = 0.8; main effect of treatment: χ^2^ = 3.12, df = 2, *P* = 0.2).

## Discussion

Our results indicate that in our intact sites in African rainforests, effects including competition for space or other resources play a role in shaping the seedling community, but in defaunated forests the lack of large seed dispersers leads to dispersal limitation becoming the dominating driving force. We conclude this based on the differences between cleared and control plots, which show that competition from standing seedlings affects germination and mortality, but this is less important for structuring differences in recruitment among plant species of different dispersal modes between hunted and protected sites (Figs 1 and 2). Thus, hunting alters which processes are important for seedling regeneration. These conclusions corroborate predictions and results from previous studies from tropical rainforests (Terborgh 2013; Culot *et al.* 2017).

However, competition among species of different dispersal modes (particularly between primate-dispersed and abiotically dispersed species) could not explain the difference in germination among species in the cleared plots between hunted and protected sites. Many fewer abiotically dispersed seedlings germinated in the cleared plots in protected sites in comparison to the cleared plots in the hunted sites. Thus, defaunation could lead to the competition–colonization trade-off (Tilman 1994) becoming much more pronounced. In an intact forest, both the large-seeded primate-dispersed seeds, and the small-seeded abiotically dispersed seeds are dispersed away from parent trees to places where they have higher chance to survive, but the large-seeded primate-dispersed species are stronger competitors. Consequently, primate-dispersed species dominate the intact forests both in terms of number of standing trees and number of species (Effiom *et al.* 2013), as primates help the competitive seeds disperse despite their size. However, in a hunted forest those trees that are dispersed by primates do not get dispersed to safe sites away from parent trees, which in effect creates a trade-off between competition and dispersal ability, which in consequence changes the patterns of seedling recruitment.

Results from the selectively cleared plots in protected sites imply that seed dispersal limitation may be more relevant in structuring this seedling community than is inter-seedling competition. Germination in selectively cleared plots was very similar to that in the control plots in protected sites, but germination of abiotically dispersed species was higher in control plots in hunted sites, where large seed dispersers are absent or heavily reduced in numbers. That is, the elimination of primate-dispersed seedlings in selectively cleared plots did not enhance germination of abiotically dispersed seedlings, or any other seedlings for that matter. This result is in agreement with conclusions drawn from previous studies that the diversity and relative abundance of species in local communities may be less influenced by local processes such as competition and more affected by regional scale influences such as seed dispersal (Ricklefs 1987; Pinto *et al.* 2014).

The mature tree composition, that is the relative abundances of species of different dispersal modes, is similar between sites (Effiom *et al.* 2013) and abundances of frugivorous birds do not differ between hunted and protected forests (Effiom *et al.* 2014). The seed rain of abiotically dispersed species or species dispersed by non-primates should therefore not differ between hunted and protected sites (Effiom *et al.* 2013). Thus, one possible additional reason why we found more abiotically dispersed seedlings in hunted sites is that the character of seed predation differs between hunted and protected forests (Effiom *et al.* 2013; Rosin and Poulsen 2016). If indeed fewer primate-dispersed seeds fall on the ground away from parent trees, where we have counted the seedlings, then the remaining such seeds will have fallen just below the parent trees. In such aggregations they would be very valuable resource for seed predators (Rosin and Poulsen 2016), which might explain the higher abundance of seed predators in those sites (Effiom *et al.* 2013, 2014). In response to the ample availability of primate-dispersed seeds under parent trees they might then switch their feeding efforts away from the seeds of other dispersal modes, which are often smaller (Galetti *et al.* 2015; Rosin and Poulsen 2016). In other words, the uneven harvest of large-bodied frugivores potentially releases rodents from competition for resources with larger mammals and allowing them to expand their dietary breadth (Galetti *et al.* 2015) towards the large and aggregated primate-dispersed seeds. Thus, it is possible that the seed predators, despite being more numerous, feed less on abiotically dispersed seeds as they have a more profitable alternative in the primate-dispersed seeds. In such a case, small-seeded, abiotically dispersed species are relieved of high mortality in the seed and early seedling stage in hunted sites with high populations of seed predators (Alvarez-Clare and Kitajima 2009). This is then an example of apparent competition between primate-dispersed and abiotically dispersed seeds, as they share a common predator (Holt 1977).

We found relatively small differences in survival of seedlings between dispersal modes and hunted and protected sites, despite different densities of seedlings of all kinds. Still the higher survival among primate-dispersed seedlings in cleared plots, compared to other dispersal modes, may point to the contribution of a growth–mortality trade-off in colonization and competitive ability between species (Tilman 1994), in structuring plant communities. Moreover, a generally larger seed size of primate-dispersed species, compared to smaller-seeded abiotically dispersed species, should give them higher establishment success, seedling survival and seedling growth rate (Moles and Westoby 2004).

We conclude that the decimation of large frugivorous seed dispersers exaggerates the effects of dispersal limitation and there are few if any processes to compensate for this loss in the regeneration of fruiting trees.

## Sources of Funding

This study was supported by grant SWE-2011-030 from Sida to O.O., grants from Kungliga Fysiografiska Sällskapet to E.O.E. and by grants from Formas to O.O. and H.G.S.

## Author contributions

O.O., G.N.I. and E.O.E. conceived and planned the study with input from H.G.S. and U.O. E.O.E. conducted fieldwork with help from O.O. O.O. performed the statistical analyses with input from E.O.E. and H.G.S. O.O., E.O.E., and G.N.I. wrote the manuscript, with input from H.G.S. and U.O.

## Conflict of Interest

None declared.
